# Application of Telemedicine Services Based on a Regional Telemedicine Platform in China From 2014 to 2020: Longitudinal Trend Analysis

**DOI:** 10.2196/28009

**Published:** 2021-07-12

**Authors:** Fangfang Cui, Xianying He, Yunkai Zhai, Minzhao Lyu, Jinming Shi, Dongxu Sun, Shuai Jiang, Chenchen Li, Jie Zhao

**Affiliations:** 1 National Engineering Laboratory for Internet Medical Systems and Applications The First Affiliated Hospital of Zhengzhou University Zhengzhou China; 2 School of Management Engineering Zhengzhou University Zhengzhou China; 3 School of Electrical Engineering and Telecommunications University of New South Wales Sydney Australia

**Keywords:** telemedicine, regional telemedicine service platform, remote consultation, efficiency, satisfaction degree, telehealth, mobile health, mHealth, remote, China

## Abstract

**Background:**

Telemedicine that combines information technology and health care augments the operational model of traditional medical services and brings new opportunities to the medical field. China promotes telemedicine with great efforts, and its practices in the deployment of telemedicine platforms and delivery of services have become important references for the research and development in this field.

**Objective:**

Our work described in this paper focuses on a regional telemedicine platform that was built in 2014. We analyzed the system design scheme and remote consultations that were conducted via the system to understand the deployment and service delivery processes of a representative telemedicine platform in China.

**Methods:**

We collected information on remote consultations conducted from 2015 to 2020 via the regional telemedicine platform that employs a centralized architectural system model. We used graphs and statistical methods to describe the changing trends of service volume of remote consultation, geographical and demographic distribution of patients, and waiting time and duration of consultations. The factors that affect consultation duration and patient referral were analyzed by multivariable linear regression models and binary logistic regression models, respectively. The attitudes toward telemedicine of 225 medical practitioners and 225 patients were collected using the snowball sampling method.

**Results:**

The regional telemedicine platform covers all levels of medical institutions and hospitals in all 18 cities of Henan Province as well as some interprovince hospitals. From 2015 to 2020, 103,957 remote medical consultations were conducted via the platform with an annual increasing rate of 0.64%. A total of 86.64% (90,069/103,957) of medical institutions (as clients) that applied for remote consultations were tier 1 or 2 and from less-developed regions; 65.65% (68,243/103,945) of patients who applied for remote consultations were aged over 50 years. The numbers of consultations were high for departments focusing in the treatment of chronic diseases such as neurology, respiratory medicine, and oncology. The invited experts were mainly experienced doctors with senior professional titles. Year of consultation, tier of hospital, consultation department, and necessity of patient referral were the main factors affecting the duration of consultations. In surveys, we found that 60.4% (136/225) of medical practitioners and 53.8% (121/225) of patients had high satisfaction and believed that telemedicine is of vital importance for the treatment of illness.

**Conclusions:**

The development of telemedicine in China shows a growing trend and provides great benefits especially to medical institutions located in less developed regions and senior citizens who have less mobility. Cases of remote consultations are mainly for chronic diseases. At present, the importance and necessity of telemedicine are well recognized by both patients and medical practitioners. However, the waiting time needs to be further reduced to improve the efficiency of remote medical services.

## Introduction

Medical services are of vital importance for the community. In China, the unbalanced distribution of health care resources has become an urgent problem [1-4]. Medical resources between hospitals are not effectively shared. High-quality services are mainly offered in capital cities and developed regions, while patients from less-developed regions (eg, rural areas) may not receive treatments responsive to their needs. In addition, the limited resources provided by less capable hospitals may not fully satisfy the needs of patients with intractable and rare diseases. Some patients may visit large hospitals that provide better services. As a consequence, patients are under more financial pressure [5], and resources from small hospitals may not be properly used. Generally speaking, low-tier hospitals usually do not have the capability to provide high-quality medical services, while large hospitals in developed cities are overloaded with patients.

To address those pain points, in recent years there has been collaboration of Chinese medical services using the internet and information technology [6-8]. Such emerging service models provide solid supports for collaborations between medical institutions. For example, regional telemedicine platforms such as the Golden Health telemedicine network and the People’s Liberation Army telemedicine network [9] have been deployed in many provinces and serve medical institutions at all levels [10,11], which significantly improves the service quality of primary hospitals and eases the pressure of overloading service volume on large hospitals. After the outbreaks of COVID-19, telemedicine platforms have been adopted by a large number of hospitals to conduct remote consultations (ie, teleconsultations), treatments, and ward rounds that reduce contacts between medical practitioners and patients [12-14].

On a global scale, telemedicine connects medical service providers in sparsely populated areas. Large or specialized hospitals offer remote consultations, diagnoses, and treatments to medical institutions and patients in less-developed regions [15]. In the early stage, telemedicine services were often performed using visual telephone, email, or Integrated Services Digital Network [16]. Later, conferencing software such as voice over Internet Protocol or FaceTime was used. In recent years, private medical networks and computing platform technologies gradually became the major enabling channels for telemedicine. In Albania, an open and shared telemedicine collaboration platform that has been widely used in Europe uses open-source technology and establishes connections via the internet [17]. Crespo et al [18] designed a telemedicine platform that provides remote health care services for elderly patients with chronic obstructive pulmonary disease. Beer et al [19] performed effective remote diagnosis and treatment of skin diseases through their telemedicine platform during the outbreak of COVID-19. In 2007, a regional telemedicine center that enables sharing of digital health care records and remote consultations between medical institutions was established in Gansu Province [10]. The West China Hospital has established a medical information platform that serves western China through digital networks and video equipment [11].

Telemedicine platforms achieve effective real-time collaborations between medical institutions from different regions or countries and cover areas including pediatric problems [20,21], skin diseases [22], neurosurgery [23], and diabetes [24]. Therefore, the service volume of telemedicine has increased dramatically. From 2005 to 2012, a state tertiary pediatric hospital in Western Australia provided remote treatments to 1312 children with burns [25]. The University of Rochester has established a telemedicine platform for the treatment of mental illness that conducts about 2000 telepsychiatric consultations per year [26]. In the United States, 15 million people were supported by telemedicine in 2015, an increase of 50% compared with the number in 2013 [27]. In China, the average number of remote consultations from hospitals that provide telemedicine services reached 714 cases per hospital in 2018 [28].

Existing research works mainly focus on the development of telemedicine platforms or analysis of medical techniques (eg, artificial intelligence–assisted diagnosis) toward a certain type of disease. However, to the best of our knowledge, few works analyze the process of telemedicine services to study how to improve the efficiency of services and satisfaction of involved parties (eg, medical practitioners and patients), critical for the optimization of telemedicine applications. In this paper, we described our research and focus on valuable insights obtained from the largest regional telemedicine platform in China, which connects more than 1000 registered medical institutions across the country. First, we introduced the design principle and choices of the platform. Second, we comprehensively analyzed the volume, process, and effectiveness of remote medical services supported by the platform. Our results provide important references for the optimization of service processes, increase in efficiency, and enhancement of service value.

## Methods

### Design of the Telemedicine Platform

The regional telemedicine platform is designed to be compatible with multiple network access methods. It connects 1037 medical institutions at the provincial, municipal, county, and township levels and provides telemedicine services to medical institutions not only in Henan Province but also in other provinces of China (Multimedia Appendix 1). The platform provides services between hospitals (ie, business-to-business); it does not provide connections between medical institutions and patients at home (ie, business-to-consumer). Its capabilities include supporting communications via video terminal devices of diverse types, facilitating data sharing among medical institutions at all levels, and enabling remote medical services such as consultation and diagnosis. The platform is implemented using a centralized system architecture with modular design, where the central module (ie, the core of our system and information exchange) mainly contains the medical information management block and the Session Initiation Protocol service block. For information exchange, the platform uses web services and visualization of databases to achieve real-time sharing of medical data between the telemedicine center and connected hospitals, which makes it convenient for medical practitioners to view health records of patients during a consultation. For resource management, the platform adopts an Internet Protocol–based multimedia system that centrally manages resources of telemedicine networks, patients, medical practitioners, and services so that partner institutes are able to access from networks with heterogeneous types. The consultation and diagnosis systems that operate on the top of the platform are implemented using browser/server architecture, which supports common operating systems such as Windows, MacOS, and Linux. Using web terminals, platform managers can perform regular maintenance of key information including registered hospitals and medical experts. Furthermore, the invited experts can view electronic medical records and communicate with the host doctors via Session Initiation Protocol video conferencing.

### Data Collection

We collected data from all medical cases that were consulted through the telemedicine platform from January 2015 to December 2020. Our dataset contains the geolocation of hospitals applied for remote consultations, gender and age information of patients, waiting times, consultation durations, withdrawals of consultation, and advice on patient referrals after teleconsultations. To guarantee the validity and effectiveness of our collected data, qualitative information on patients, medical practitioners, and consultation results is approved by all participants before being formally recorded. Quantitative data such as waiting time and consultation duration are automatically measured by the platform to avoid human error. We note that the waiting time is the period between an application being submitted by a partner hospital and the start time of the consultation. The consultation duration is the effective communication period between all involved parties of a consultation. We performed data cleansing and preprocessing to obtain a structured dataset that facilitates our analysis. In addition, to understand the effectiveness of remote consultations, we collected information on the attitudes toward each consultation from the participating patients and medical practitioners. To be more specific, we selected one representative medical institution in each city that is covered by our platform to conduct our survey. Both patients and medical practitioners were invited through the snowball sampling method. As the final step of their teleconsultation process, all invited participants answered our survey. From patients, we collected their opinions on level of satisfaction toward the consultation process and results, benefits of remote consultation in reducing financial costs, and increased convenience. We collected attitudes from medical practitioners regarding each consultation process and its effectiveness.

### Statistical Analysis

During this study we collected data from 103,957 remote consultation cases. Numerical indicators such as quantity, average value, and composition ratio were used in our descriptive analysis. The quantitative data were described by the mean values, while the qualitative data were described by the counts and percentages. We calculated the average annual growth rate of teleconsultation encounters. With the support of Excel (Microsoft Corp) software, we drew line charts to describe the changing trends of consultation cases over time. Using SPSS (version 23.0, IBM Corp) software, we employed multivariable linear regression to analyze the impact of the information of applicant hospitals, patients, and consultations on consultation durations. Binary logistic regression analysis was used to understand the impact of the rank of applicant hospital, geolocation, patient status, consultation departments, and other factors on the referral recommendations. In our statistical tests, the significance levels were set to α=.05. Incorrect and incomplete data were treated as missing values.

## Results

### Regional Telemedicine Platform

**Figure 1:**
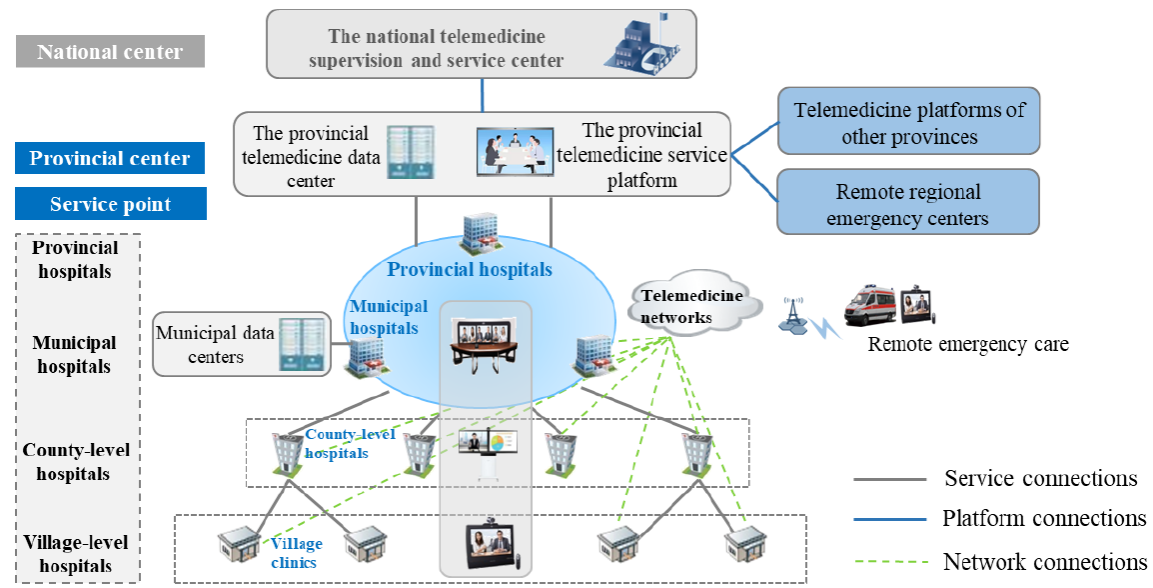
Overall connectivity of the regional telemedicine platform.

The regional telemedicine platform connects medical institutions at all levels including provincial hospitals, municipal hospitals, county-level medical institutions, township health centers, community service centers, and village-level clinics. Figure 1 shows the overall connectivity scheme of the platform. Communications between each institution are established via virtual private medical networks that enable service collaboration and health data sharing across hospitals. Apart from the provincial center, municipal telemedicine centers are deployed in 18 cities of the Henan Province. Telemedicine centers of both levels (ie, provincial and municipal) have their own dedicated medical data centers, which together form a dual-active data center. Medical data that are used by and generated from each consultation are stored in the provincial data center and copied to the corresponding municipal data center. Medical institutions at various levels select different types of video conferencing equipment according to their needs and available resources. For example, provincial and municipal hospitals mainly use large-scale multiscreen consultation terminals with the highest audio and video quality, county-level institutions usually choose dual-screen separate consultation terminal devices, and township health centers and village-level clinics are likely to use single-screen computers with embedded cameras and microphones as their consultation terminals. The regional telemedicine platform is connected with the regional emergency center and telemedicine platform in other provinces via virtual private medical networks. In addition, mobile ambulances are connected to the platform through 5G networks. A registered hospital can initiate a consultation by logging onto the platform to provide information on patients, invite participants, and submit an application. The invited hospital schedules the consultation and informs the corresponding medical practitioners. All participants join in the teleconsultation using terminal devices. Results of each consultation are automatically recorded and maintained by the system for future reference.

### Teleconsultation Service Volume and Characteristics

**Figure 2:**
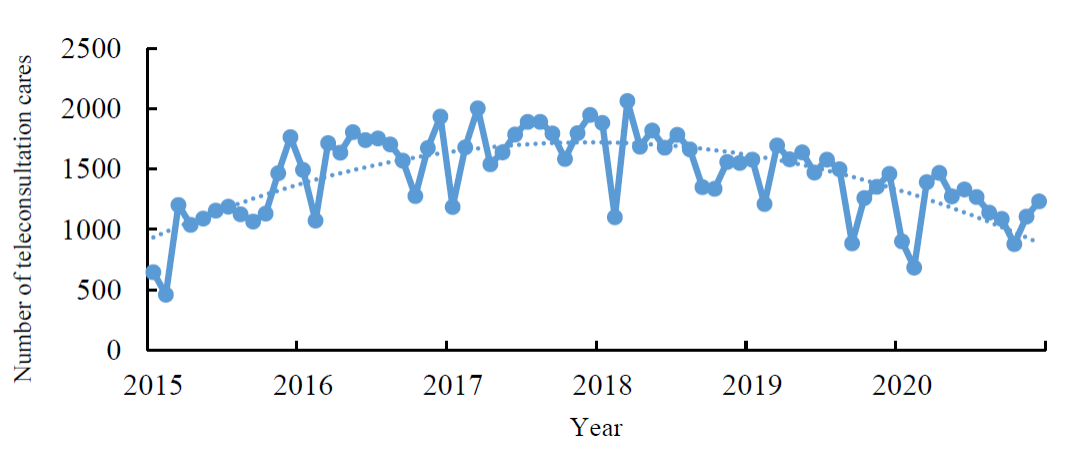
Changing tendency of service volume from 2015 to 2020.

From 2015 to 2020, 103,957 remote medical consultations were conducted through the telemedicine platform. There were 12.83% (13,337/103,957), 18.65% (19,391/103,957), 19.96% (20,753/103,957), 18.74% (19,480/103,957), 16.57% (17,225/103,957), and 13.25% (13,771/103,957) teleconsultation encounters in the years 2015, 2016, 2017, 2018, 2019, and 2020, respectively (Multimedia Appendix 2). There was an overall increasing trend with an annual rate of 0.64%. We observed fluctuations in the patterns in different months. The number of teleconsultation encounters was high in November and December and relatively low in January and February (Figure 2). That is, the service volume was large in the winter but smaller during Lunar New Year (ie, January and February).

**Figure 3:**
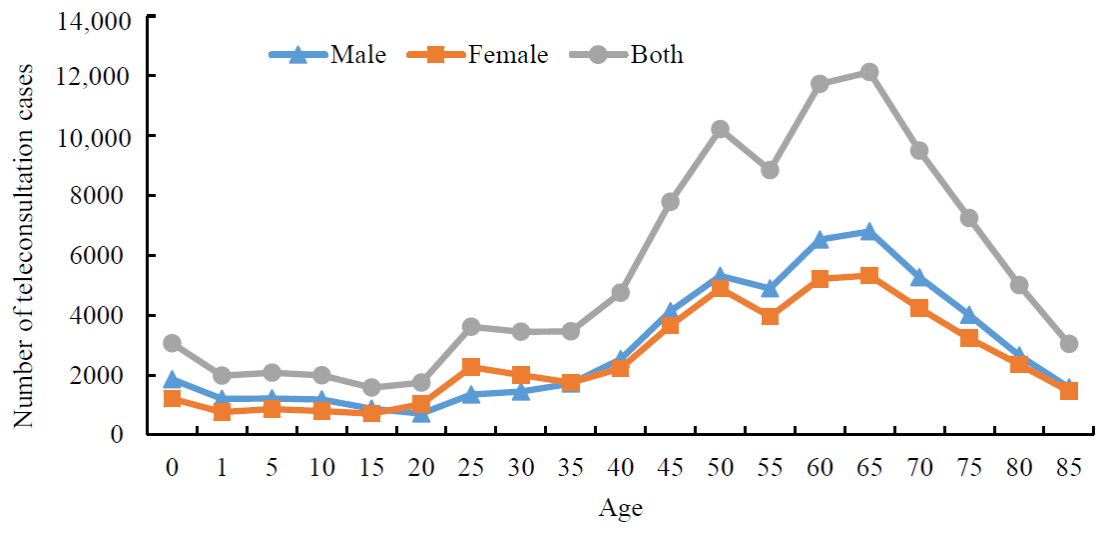
Distributions of gender and age of patients served.

Considering the characteristics of patients who participated in teleconsultation encounters, 53.55% (55,664/103,945) were male. The majority (68,243/103,945, 65.65%) were aged over 50 years, and patients aged from 65 to 70 years corresponded to the largest group with the total fraction of 11.76% (12,229/103,945; Figure 3). The geolocation of patients was labeled as either intraprovince or interprovince. From 2015 to 2020, 98.70% (102,607/103,957) of teleconsultation encounters were labeled intraprovince, and the average annual increasing rate for this group was 0.41%; 1.30% (1350/103,957) of patients were interprovince, with an average annual increasing rate of 21.27%. We noted that patients served by the telemedicine platform were mainly within the province (ie, Henan Province), but the service volume for interprovince cases increased significantly. There are 18 cities in Henan Province. When we zoom in on the group of patients within the province, a large proportion of them were from several cities including Nanyang, Pingdingshan, and Zhengzhou.

Hospitals that applied for remote consultations contain all tiers (ie, tier 1, 2, and 3, where tier 3 hospitals have the highest ranks and the best medical resources). While 86.64% (90,069/103,957) of consultations were requested by tier 1 and tier 2 hospitals, only 13.36% (13,888/103,957) of cases were for tier 3 hospitals (Multimedia Appendix 2). Although the majority of applicants were tier 2 hospitals, the number of applications from tier 3 hospitals rapidly increased from 2015 to 2020 with an average annual rate of 47.12%. On the contrary, the changing rate for the group of tier 1 and tier 2 hospitals was –2.87%, which shows a dropping trend. As for the departments that applied for teleconsultation, internal medicine departments requested the majority (66,970/103,597, 64.42%) of cases, and 24.33% (25,295/103,597) of consultations were requested by surgery departments. Other disciplines included gynecology, pediatrics, and otolaryngology; the top three departments were neurology, respiratory medicine, and pediatric internal medicine. Table 1 shows the number of teleconsultations requested by each department and their percentage values for each year. Top 10 departments were mapped to 63.97% (66,276/103,597) of cases. Almost all (102,960/103,957, 99.04%) medical practitioners and experts from invited hospitals had senior professional titles (eg, professor).

**Table 1:** Top 10 departments applied for teleconsultation.

Department	2015, n (%)	2016, n (%)	2017, n (%)	2018, n (%)	2019, n (%)	2020, n (%)
Neurology	1834 (13.75)	2869 (14.80)	2985 (14.38)	2263 (11.62)	1935 (11.23)	1444 (10.49)
Respiratory medicine	1283 (9.62)	1898 (9.79)	2499 (12.04)	2643 (13.57)	2368 (13.75)	2122 (15.41)
Pediatric internal medicine	962 (7.21)	1199 (6.18)	1422 (6.85)	1570 (8.06)	1329 (7.72)	888 (6.45)
Oncology	764 (5.73)	1117 (5.76)	1291 (6.22)	1273 (6.53)	1083 (6.29)	955 (6.93)
Orthopedics	792 (5.94)	1206 (6.22)	1197 (5.77)	1064 (5.46)	1035 (6.01)	646 (4.69)
Cardiovascular medicine	850 (6.37)	885 (4.56)	1058 (5.10)	909 (4.67)	894 (5.19)	696 (5.05)
Neurosurgery	615 (4.61)	936 (4.83)	868 (4.18)	841 (4.32)	817 (4.74)	660 (4.79)
Gastroenterology	488 (3.66)	716 (3.69)	680 (3.28)	682 (3.50)	536 (3.11)	439 (3.19)
Hepatobiliary, pancreatic and liver transplantation	502 (3.76)	704 (3.63)	651 (3.14)	601 (3.09)	536 (3.11)	431 (3.13)
Gynecology	449 (3.37)	653 (3.37)	628 (3.03)	599 (3.07)	623 (3.62)	393 (2.85)

### Teleconsultation Service Process

The waiting time between an application submitted by the applicant hospital and the start of the teleconsultation care reflects the efficiency of a case. Results from our analysis showed that the median value of waiting time was 24.27 hours, the minimum value was 11.00 minutes, and the maximum value was 7.00 days (Multimedia Appendix 2). From 2015 to 2020, the waiting time in different years appeared to be statistically different (rank sum test [H]=8309.00, *P*<.001). The median value for the year 2015 was 15.92 hours, while the value for the year 2020 was 24.27 hours. We observed an increasing trend in years for the waiting time. While the service capability in provincial hospitals remained almost unchanged, as our platform was promoted, more cases (especially those involving rare and intractable diseases) were diagnosed through teleconsultation encounters. As a consequence, the average waiting time increased.

The consultation duration between the start and end of teleconsultation reflects the quality and complexity of the service process to some extent. From 2015 to 2020, the median value was 17.00 minutes (Multimedia Appendix 2)—18.00 minutes in 2016 and 26.00 minutes in 2020. We noted that the consultation duration also increased among years statistically (H=3072.93, *P*<.001). The consultation duration was affected by many factors such as participating disciplines, condition of patients, and capability of medical practitioners. Therefore, we performed multivariable linear regression to analyze the influence of those key factors (eg, application content and patients) on consultation duration. In our regression analysis, consultation duration is the dependent variable. Independent variables contain consultation date, level (or tier) of the applicant hospital, gender of the patient, age of the patient, discipline (or department), title of the medical practitioner, and waiting time. Results show that independent variables such as year of consultation, applicant hospital level, and department were considered as significant inputs by the model (as shown in Table 2). Compared with the value in 2015, the average consultation duration increased in 2018, 2019, and 2020, with β values of 4.13, 3.08, and 8.16, respectively (*P*<.001). Consultation durations are usually longer when applicant institutes are tier 3 hospitals (β=1.69, *P*<.001) or patients have been referred (β=0.69, *P*=.01). Consultation durations for surgical departments are shorter than those for internal medicine departments (β=–1.00, *P*<.001).

**Table 2:** Results of multivariable linear regression analysis for impact factors of consultation durations.

Variable			β (95% CI)		SE		T score		*P* value
Constant			21.04 (19.70 to 22.37)		0.68		30.96		<.001
**Year (ref^a^: 2015)**									
	2016	–4.00 (–4.36 to –3.64)		0.18		–21.93		<.001	
	2017	0.25 (–0.15 to 0.65)		0.21		1.23		.22	
	2018	4.31 (3.66 to 4.61)		0.24		17.10		<.001	
	2019	3.08 (2.58 to 3.59)		0.26		12.03		<.001	
	2020	8.16 (7.60 to 8.73)		0.29		28.40		<.001	
**Applicant hospital level (ref: tier 2 and below hospital)**									
	Tertiary	1.69 (1.29 to 2.09)		0.2		8.31		<.001	
Waiting time			0.00 (0.00 to 0.00)		0		1.85		.06
Transfer treatment (ref: no)			0.69 (0.17 to 1.21)		0.27		2.6		.01
**Consultation department (ref: internal medicine department)**									
	Surgery department	–1.00 (–1.30 to –0.71)		0.15		–6.63		<.001	
	Otolaryngology department	0.58 (–0.11 to 1.26)		0.35		1.64		.10	
	Gynecology & pediatrics departments	–0.92 (–1.47 to –0.36)		0.28		–3.25		.001	
	Medical technology department	0.28 (–0.49 to 1.05)		0.39		0.71		.48	
**Title of invited consultant (ref: attending doctor)**									
	Associate chief physician	–0.78 (–2.09 to 0.54)		0.67		–1.16		.25	
	Chief physician	–1.51 (–2.83 to –0.19)		0.67		–2.25		.03	

^a^ref: reference.

### Analysis of Withdrawal and Referral Cases

From 2015 to 2020, 5.09% (5270/103,957) of cases were withdrawn before the start of teleconsultation. Major reasons included the patient having been transferred (1209/5270, 22.94%) and the applicant medical practitioner requested a withdraw of consultation (1310/5270, 24.86%). This work investigates the characteristics of referral cases since 2017. After a teleconsultation, the invited medical experts may give referral advice accordingly. A referral decision is made by considering a collection of factors such as severity of condition, physical distance (ie, within or outside Henan Province), and level of resident hospital. Therefore, we analyzed the impact of different factors on the referral cases. From 2017 to 2020, 15.27% (10,878/71,299) of cases received suggestions for referral from invited medical experts after consultations. We used binary logistic regression analysis to understand relations between those suggestions of referral and factors such as level of applicant hospitals, conditions of patients, and participated departments. From our results, we have reached 3 key observations. First, patients within Henan Province were more likely to receive suggestions of referral compared with those who were in other provinces (OR 2.17, *P*<.001). Second, older patients had a low referral rate (OR 1.00, *P*=.002). Third, compared with consultations hosted by the internal medicine department, those by surgery department, otolaryngology department, and gynecology and pediatrics departments had higher rates of referrals, with *P*<.001 and OR values of 2.94, 4.53, 3.51, respectively (Table 3).

**Table 3:** Results of binary logistic regression analysis for impact factors of referral cases.

Variable			β	SE		Wald		OR^a^ (95% CI)		*P* value
Constant			–4.98	0.30		274.24		0.007		*<*.001
**Region (ref ^b^: other provinces)**										
	Henan province	0.77		0.22	12.34		2.17 (1.41-3.33)		*<*.001	
**Applicant hospital level (ref: tier 2 and below hospital)**										
	Tertiary	0.35		0.06	39.94		1.41 (1.27-1.57)		*<*.001	
Age			–0.003	0.001		9.58		1.00 (1.00-1.00)		.002
**Consultation department (ref: internal medicine department)**										
	Surgery department	1.08		0.20	29.88		2.94 (2.00-4.32)		*<*.001	
	Otolaryngology department	1.51		0.20	58.18		4.53 (3.07-6.67)		*<*.001	
	Gynecology & pediatrics departments	1.26		0.22	33.22		3.51 (2.29-5.37)		*<*.001	
	Medical technology department	1.43		0.21	47.45		4.12 (2.79-6.30)		*<*.001	
**Title of invited consultant (ref: attending doctor)**										
	Associate chief physician	0.49		0.17	8.34		1.63 (1.17-2.27)		.004	
	Chief physician	0.07		0.04	3.25		1.07 (1.00-1.16)		.07	
Consultation time			0.02	0.001		256.81		1.02 (1.02-1.02)		*<*.001

^a^OR: odds ratio.

^b^ref: reference.

### Attitudes Toward Telemedicine From Medical Practitioners and Patients

From our investigations into attitudes toward telemedicine from medical practitioners, we have observed that the majority of them held positive views; 68.4% (154/225) of participating experts believed that telemedicine is of great help in increasing the service quality of medical practitioners and 59.1% (133/225) believed that telemedicine is of great help in reducing financial burdens on patients. Considering the level of satisfaction toward consultation processes, 60.4% (136/225) of medical practitioners were very satisfied, and 76.9% (173/225) would recommend telemedicine services for their patients (Table 4). Patients also showed positive attitudes toward the telemedicine services they have received, with 60.0% (135/225) saying telemedicine is helpful for treatment of their disease, and 62.2% (140/225) agreeing that remote consultations provide convenience. In addition, 53.8% (121/225) were very satisfied with telemedicine services, and 59.1% (133/225) would suggest telemedicine to other patients (Table 5).

**Table 4:** Attitudes toward telemedicine from medical practitioners.

Item			Response, n (%)
**Do you believe that telemedicine is helpful in improving the service quality of medical practitioners?**			
	Very helpful	154 (68.4)	
	Helpful	70 (31.1)	
	Not helpful	1 (0.4)	
**Do you believe that telemedicine can help to reduce the financial burden on patients?**			
	Very helpful	133 (59.1)	
	Helpful	85 (37.8)	
	Not helpful	7 (3.1)	
**What is your level of satisfaction with the service process of telemedicine?**			
	Very satisfied	136 (60.4)	
	Satisfied	88 (39.1)	
	Not satisfied	1 (0.4)	
**Would you like to participate in telemedicine services in the long term?**			
	Very likely	177 (78.7)	
	Likely	45 (20.0)	
	Not likely	3 (1.3)	
**Would you recommend telemedicine services for patients?**			
	Very likely	173 (76.9)	
	Likely	49 (21.8)	
	Not likely	3 (1.3)	

**Table 5:** Attitudes toward telemedicine from patients.

Item		Response, n (%)
**Do you believe that telemedicine is helpful for your treatments?**		
	Very helpful	135 (60.0)
	Helpful	89 (39.6)
	Not helpful	1 (0.4)
**Do you believe that telemedicine reduces the financial cost of seeking medical treatment?**		
	Yes	215 (95.6)
	No	2 (0.9)
	Not sure	8 (3.6)
**Does telemedicine provide convenience?**		
	Very convenient	140 (62.2)
	Convenient	83 (36.9)
	Not convenient	2 (0.9)
**Are you satisfied with the service process of telemedicine?**		
	Very likely	133 (59.1)
	Likely	91 (40.4)
	Not likely	1 (0.4)

## Discussion

### Principal Findings

In recent years, telemedicine has been promoted rapidly in China. Many governmental policies and documents requesting the promotion of telemedicine to townships and rural areas have been issued. A large scope of services such as teleconsultation, telepathology, tele-electrocardiogram, and telediagnosis of medical images are widely offered with the business-to-business service mode. The current progress in telemedicine has greatly improved the service quality of primary medical institutions and brought convenience to patients. We note that further improvements in consultation efficiency and optimization of the consultation process are yet to be addressed. In this paper, we investigated the first and largest regional and comprehensive telemedicine platform deployed in China that changed legacy medical consultation methods (ie, via telephone and communication software) by realizing telemedicine services through platform technologies. It is a representative milestone toward the promotion of telemedicine in China. The platform that is supported by the largest hospital in Henan Province offers teleconsultation services to medical institutions at all levels within and outside the province. Since 2015, the service volume of this regional platform showed an increasing pattern followed by a slowly decreasing trend. With the adoption of telemedicine services by more medical institutions, municipal and county-level hospitals can host remote consultations for primary medical institutions. Therefore, teleconsultation cases for common diseases are offloaded to municipal or county-level hospitals that are more responsive than large institutions. During the pandemic of COVID-19, the patients with normal (or chronic) conditions preferred to defer their visits to hospitals, and the number of hospitalized patients at all levels of institutions decreased significantly. Thus, we saw a decreasing pattern of service volume for this provincial platform from 2018 to 2020. We note that the frequent connections between medical institutions at adjacent levels is one objective of the hierarchical medical system in China.

From 2015 to 2020, 103,957 teleconsultations were conducted through this platform. The majority of patients were from Henan Province with a much smaller percentage of interprovince patients, which is consistent with the results of existing studies [29]. Patients participating in remote consultations were mostly aged over 50 years, with higher risks in having diseases [30,31]. Applicant institutions were mainly tier 1 or 2 hospitals, defined as primary hospitals with limited medical capability, especially in the treatment of severe, intractable, and rare diseases [32]. Therefore, they need supports from large hospitals through the telemedicine platform. Our study also revealed the popular disciplines involved in teleconsultation encounters, including neurology, respiratory medicine, oncology, and cardiovascular medicine. As we know, the spectrum of human diseases has changed with the development of society. Chronic diseases such as stroke, malignant tumor, chronic obstructive pulmonary disease, heart disease, and circulatory disease have become critical problems for the community [33-35]. As a result, the number of patients with those issues keeps increasing [36], leading to high demands for medical consultations in related disciplines. To guarantee service quality, it is suggested by most provincial governments that the invited experts for medical consultations should have senior professional titles. In our study, we found that 99.04% of invited doctors met the requirements, which is much higher than the percentage of legacy face-to-face consultations. Only 27.5% of doctors in primary medical institutions (eg, township hospitals and village clinics) have proper professional titles, which is one of the reasons that they could not provide high-quality medical services [37,38]. As one motivation for the rapid development of telemedicine, patients of primary hospitals could also receive better diagnoses and treatments from experienced experts with senior titles.

Due to the large population of China, there is a high demand for medical resources. It is quite common for large hospitals that provide high-quality medical services to have an excessive number of patients, long waiting times, and degraded efficiency of medical treatments [39]. However, with the support from the telemedicine platform, the average waiting time after application submitted by applicant hospitals is 1388.00 minutes (ie, 23.13 hours), indicating that the majority of cases could be processed within a day, effectively increasing the efficiency of medical services. Some studies have reported that the average waiting time for the return of remote pathology results exceeds one day [40], and the waiting time for remote pathology consultation results in some countries exceeds 4 days [41]. In contrast, the waiting time for remote consultations supported by our platform is relatively short. The average consultation duration was 17.00 minutes. As discovered by our multivariable analysis, the consultation duration increases with time. The consultation quality increases with the advances of communication and digital conferencing technologies [42], which make it convenient for participants to have a more comprehensive and in-depth discussion. The consultation duration is longer for patients who received referrals as their conditions are rarer and more complicated.

After teleconsultation services, the invited medical experts may make referral suggestions. Compared with intraprovince patients, we found that interprovince patients are more likely to receive referral suggestions. It is mainly because that interprovince referrals are quite inconvenient due to long physical distances, and the government encourages patients to receive medical treatments within their home provinces. Elderly patients with limited mobility, for example, have a low referral rate. Since the quality of surgical operations is usually better in large hospitals, a higher referral rate is observed for cases in surgery departments.

As we found in our study, the majority of medical practitioners believe that telemedicine improves service qualities and they would like to participate in teleconsultation services, consistent with existing research findings [43,44]. The promotion of telemedicine is beneficial for doctors on both sides. Through remote consultations, the invited experts have more opportunities to gain experiences on intractable and rare diseases, while doctors from the applicant hospitals learn from their counterparts at large hospitals. Prior research works have shown that the overall satisfaction of doctors in traditional medical service processes is moderate, and the personal satisfaction of doctors is quite low [45,46]. However, we illustrated that 60.4% of medical practitioners experienced a high level of satisfaction with the process of telemedicine services, indicating its popularity among doctors.

Most doctors and patients agreed that the deployment of telemedicine could save financial costs, which is consistent with the views of existing research [47]. Some researchers found that telemedicine could help patients save US $1000, on average [48]. Higher level hospitals provide lower level medical institutions with remote consultations so that patients from less capable hospitals also receive advice from experts with senior titles, which is very beneficial for the patients in rural areas. We found that 60.0% of patients believe telemedicine is helpful in the treatment of their medical conditions, and 53.8% are very satisfied with the services they have received.

### Limitations

The paper analyzes the process of remote consultation and provides an important reference for improving service efficiency. However, the types of telemedicine services are diverse, and research on the other service types such as remote education and remote nursing needs to be further developed. In addition, the data used in this paper were collected from a regional telemedicine platform, which cannot fully represent the development of telemedicine in China, and the overall application of telemedicine services needs further exploration.

### Conclusions

In this paper, we describe the architecture and functionality of the first regional telemedicine center in China built using platform technologies. We collected and analyzed teleconsultation care services conducted through the platform from 2015 to 2020. Our work reveals the growth trend of service volume, reveals that the majority of applicant institutions are tier 2 hospitals, and shows that middle-aged and elderly patients make up the largest age group. In China, the promotion and deployment of telemedicine services is occurring at a rapid speed, which meets the urgent demand of medical institutions with less capability and the medical need of aged patients with less mobility. The efficiency of telemedicine is increasing with the advances of information technologies. Both medical practitioners and patients have high levels of satisfaction. It is believed that the deployment of a telemedicine platform not only increases the efficiency of medical consultations but also reduces the financial burdens of patients; thus, telemedicine is worthy of further promotion.

## Appendix

Multimedia Appendix 1 Distribution of the hospitals connected to the regional platform.

Multimedia Appendix 2 Basic information in the teleconsultations.
