# Supplementary figures and images for: Application of Telemedicine Services Based on a Regional Telemedicine Platform in China From 2014 to 2020: Longitudinal Trend Analysis
Source: J Med Internet Res. 2021 Jul 12;23(7):e28009. doi: 10.2196/28009 (PMC8314158; doi:10.2196/28009)

**Multimedia Appendix 1.** The distribution of the hospitals connected to the regional platform

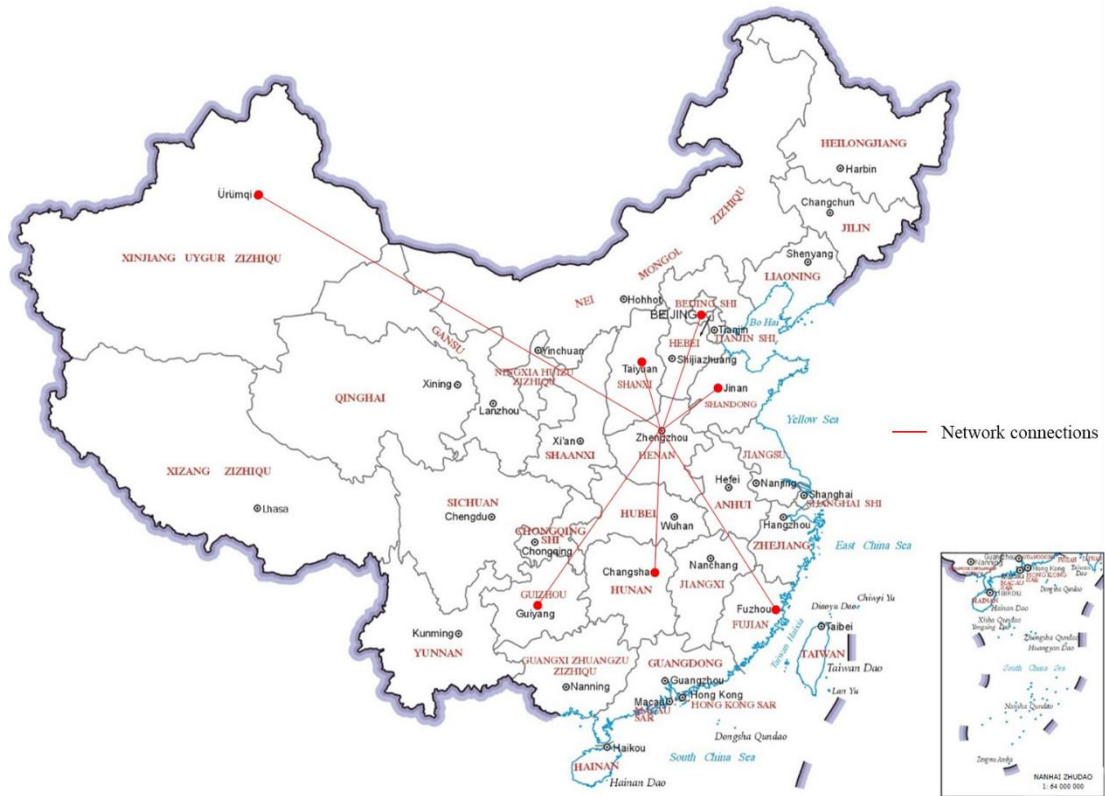

Supplement: Multimedia Appendix 1 [file jmir_v23i7e28009_app1.pdf]
