# Supplementary material for: Application of Telemedicine Services Based on a Regional Telemedicine Platform in China From 2014 to 2020: Longitudinal Trend Analysis
Source: J Med Internet Res. 2021 Jul 12;23(7):e28009. doi: 10.2196/28009 (PMC8314158; doi:10.2196/28009)
Supplement: Multimedia Appendix 2 [file jmir_v23i7e28009_app2.pdf]

Multimedia Appendix 2. The basic information of the teleconsultations

Month	Teleconsultations for patients of different genders			Teleconsultations applied by hospitals of different tiers		Median value of waiting time (hours)	Median value of consultation duration (minutes)	Numbers of withdrawn cases
	Total	Male	Female	Tier-3 hospitals	Tier-2 and tier-1 hospitals			
2015	13337	7097	6240	372	12965	15.92	18.00	91
2016	19391	10488	8903	2003	17388	19.30	13.00	1033
2017	20753	11061	9692	2910	17843	23.82	16.00	1186
2018	19480	10459	9021	3123	16357	24.73	21.00	1069
2019	17225	9121	8104	2916	14305	24.27	21.00	1055
2020	13771	7445	6326	2564	11201	24.27	26.00	836
Total	103957	55671	48286	13888	90059	23.13	17.00	5270
